# 5-Methyl-Tetrahydrofolate and the S-Adenosylmethionine Cycle in C57BL/6J Mouse Tissues: Gender Differences and Effects of Arylamine N-Acetyltransferase-1 Deletion

**DOI:** 10.1371/journal.pone.0077923

**Published:** 2013-10-25

**Authors:** Katey L. Witham, Neville J. Butcher, Kim S. Sugamori, Debbie Brenneman, Denis M. Grant, Rodney F. Minchin

**Affiliations:** 1 School of Biomedical Sciences, University of Queensland, Brisbane, Queensland, Australia; 2 Department of Pharmacology and Toxicology, University of Toronto, Toronto, Ontario, Canada; The Walter and Eliza Hall of Medical Research, Australia

## Abstract

Folate catabolism involves cleavage of the C^9^-N^10^ bond to form p-aminobenzoylgluamate (PABG) and pterin. PABG is then acetylated by human arylamine *N*-acetyltransferase 1 (NAT1) before excretion in the urine. Mice null for the murine NAT1 homolog (Nat2) show several phenotypes consistent with altered folate homeostasis. However, the exact role of Nat2 in the folate pathway *in vivo* has not been reported. Here, we examined the effects of Nat2 deletion in male and female mice on the tissue levels of 5-methyl-tetrahydrofolate and the methionine-*S*-adenosylmethionine cycle. We found significant gender differences in hepatic and renal homocysteine, *S*-adenosylmethionine and methionine levels consistent with a more active methionine-*S*-adenosylmethionine cycle in female tissues. In addition, methionine levels were significantly higher in female liver and kidney. PABG was higher in female liver tissue but lower in kidney compared to male tissues. In addition, qPCR of mRNA extracted from liver tissue suggested a significantly lower level of Nat2 expression in female animals. Deletion of Nat2 affected liver 5- methyl-tetrahydrofolate in female mice but had little effect on other components of the methionine-*S*-adenosylmethionine cycle. No N-acetyl-PABG was observed in any tissues in Nat2 null mice, consistent with the role of Nat2 in PABG acetylation. Surprisingly, tissue PABG levels were similar between wild type and Nat2 null mice. These results show that Nat2 is not required to maintain tissue PABG homeostasis *in vivo* under normal conditions.

## Introduction

Folates are essential group B9 vitamins required for DNA and RNA synthesis, DNA and protein methylation, and lipid modification [1]. The major folate present in mammalian cells is 5-methyl-tetrahydrofolate (5-MTHF), which provides methyl groups to the *S*-adenosylmethionine (SAM) cycle through the conversion of homocysteine (Hcy) to methionine [2]. SAM is the common cofactor in all cellular methyltransferase reactions. Consequently, folate intake influences DNA methylation [3], [4], an essential epigenetic regulatory mechanism in cells. Dietary folate has been linked to a number of human pathologies including neural tube defects [5], orofacial clefts [6], neurological disorders [7], gastrointestinal disease [8], cancer [9] and coronary heart disease [10].

An important catabolic pathway in folate homeostasis is cleavage of the C^9^-N^10^ bond to form *p*-aminobenzoylglutamate (PABG) [11]. In humans, PABG is acetylated by arylamine *N*-acetyltransferase 1 (NAT1) to *N*-acetyl-PABG [12], which is a major folate metabolite found in the urine [13]. Genetic polymorphisms that affect NAT1 activity have been linked to folate-dependent abnormalities such as orofacial clefts [14] and spina bifida [15]. These studies suggest that NAT1 may have an important role in the maintenance of tissue folate levels.

In mice, the NAT1 homolog is termed Nat2. It has high sequence homology and substrate specificity to human NAT1 [16]. Nat2 also specifically acetylates PABG [17]. The Nat2 null (Nat2−/−) mouse has been a useful model to investigate the role of Nat2 in folate homeostasis. Wild-type animals excrete *N*-acetyl-PABG in the urine but Nat2−/− mice do not, confirming the role of Nat2 in PABG metabolism [18]. Deletion of Nat2 in some mouse strains increases the incidence of several phenotypes that may be attributed to altered folate effects. These include neural tube defects [18] and ocular defects [19]. However, the effect of Nat2 on folate tissue and blood levels, or the SAM cycle, has not been reported.

In the present study, we have compared the tissue levels of 5-MTHF and the folate catabolites in wild-type and Nat2−/− C57BL/6 mice. In addition, components of the SAM cycle were investigated and showed significant gender differences. However, the concentrations of pABG in all tissues examined was unaffected by deletion of Nat2.

## Materials and Methods

### Animal Tissues and Ethics Statement

All animal procedures were performed in accordance with Canadian Council on Animal Care (CCAC) guidelines and were approved by the University of Toronto Faculty of Medicine and Pharmacy Animal Care Committee. All procedures were performed under anesthesia. Nat2−/− mice on a congenic C57BL/6J background were originally provided courtesy of Professor Edith Sim (Oxford University), and have been maintained in a separate breeding colony at the University of Toronto. All mice were housed under standard 12-hour light/dark cycle conditions, and were provided free access to a Tekland Global 18% protein rodent diet. At 8 weeks of age, mice were anesthetized with Avertin, a sample of blood was collected, and animals were perfused with PBS to remove remaining blood. Liver, kidneys, brain, heart and lungs were excised, and tissues and blood samples were snap frozen in liquid nitrogen and stored at −80°C until analyzed.

### Sample Preparation

Approximately 50 mg of liver, kidney, brain, heart or lung tissue was sonicated in 0.5 ml of ice cold water before centrifugation at 100,000×g for 30 min at 4°C. Approximately 0.1 ml of whole blood in a final volume of 0.5 ml ice cold water was sonicated before centrifugation at 16,000×g for 10 min at 4°C.

Liquid phase extraction of the tissue/blood supernatant was carried out by adding 0.425 ml of methanol and 0.525 ml of chloroform to 0.4 ml of supernatant and 10 µl internal standard (*p*-aminohippuric acid). The aqueous phase was collected, reduced to near dryness under vacuum centrifugation and resuspended in 30 µl 2% methanol/0.1% formic acid for immediate analysis by LC-MS. Standard curves were constructed and used to calculate the concentration of each metabolite in the samples.

In order to avoid interference with other assays, reducing agents were not routinely added for the extraction of 5-MTHF. Consequently, initial studies were performed to determine whether their omission affected recovery and/or stability. Tissue homogenates prepared in ammonium acetate buffer (pH 7.4) containing 1% ascorbate and 0.1% 2-mercaptoethanol were spiked with 2.4 nmol 5-MTHF and extracted as described above. 5-MTHF and PABG levels were then determined by LC-MS/MS (see below) and compared to extraction from water. There were no differences in the measured amounts of either 5-MTHF or PABG between the different extraction procedures.

### High Pressure Liquid Chromatography

The LC-MS/MS system consisted of an Agilent 1200 Series LC system with an Agilent 1260 Series column oven (Agilent Technologies, Palo Alto, CA, USA) coupled with an API 3200 triple quadrupole mass spectrometer with electron ion source (ABSciex, Foster City, CA, USA). HPLC separation of analytes was performed on a Poroshell 120 SB-C18 column (150 mm×2.1 mm, 2.7 µm particle size, Agilent Technologies) with gradient elution comprised of 0.1% formic acid in water (eluent A) and 0.1% formic acid in methanol (eluent B). Two gradients were utilized, one to separate SAM cycle components (Gradient 1) and the other to separate PABG, polyglutamated forms of PABG and N-acetyl-PABG (Gradient 2). The flow rate for both gradients was 0.15 ml/min. The column was maintained at a constant temperature of 37°C while the autosampler tray temperature was maintained at 4°C. Gradient 1 started at 0% B (100% A) increasing to 20% B over 2 min followed by an increase to 98% B over 3 min with column flushing at 98% B for 2 min before returning to 0% B. Gradient 2 started at 2% B (98% A) increasing to 70% B over 20 min followed by an increase to 98% B over 2 min with column flushing at 98% B for 1 min before returning to 0% B.

The detection of the analytes by mass spectrometry was carried out using positive electrospray ionization and multiple reaction monitoring mode. The ion source parameters were optimized for all analytes. The ionspray voltage was set at 5500 V and the ionspray source temperature was 500°C. The interface heater was set at 100°C. The curtain gas, ion source gas 1 (nebulizer gas) and ion source gas 2 (turbo gas) were set to 30, 50 and 50 psi, respectively. Three precursor-to-product transitions for each compound were measured.

### Quantification of Tissue Nat2 mRNA

Total RNA was extracted from approximately 20 mg of liver and kidney tissue from male and female wild-type mice using an RNeasy mini kit (Qiagen, Chadstone, VIC, Australia) and cDNA was synthesized using Superscript III reverse transcriptase (Invitrogen, Mulgrave, VIC, Australia) and oligo(dT)_15_ primer (Promega, Alexandria, NSW, Australia) as per the manufacturer’s instructions. Quantitative PCR used specific primers for Nat2 (sense 5′-gcacaacacagtcctgactttcc-3′, antisense 5′-aagtccagtttgctcctggtgc-3′) or β-actin (sense 5′-cctaaggccaaccgtgaaaag-3′, antisense 5′-tcttcatggtgctaggagcca-3′) and Sensimix SYBR kit (Bioline, Alexandria, NSW, Australia) and was performed using an iCycler PCR machine (Bio-Rad, Gladesville, NSW, Australia). PCR protocol consisted of 95°C for 10 min followed by 40 cycles of 95°C for 10 s, 58°C (Nat2) or 52°C (β-actin) for 15 s, 72°C for 20 s. Melt curves also were performed to show PCR specificity. No PCR product was detected for Nat2 using cDNA from male Nat2−/− liver tissue. Results were normalized to β-actin and expressed relative to male samples using the ΔΔCT method.

### Statistical Analysis

Student’s t-test or 1-way ANOVA was used to compare metabolite concentrations in the different tissues (GraphPad Prism, Version 5.03). Significance was assumed at p<0.05.

## Results

### Tissue Levels of 5-MTHF, Hcy and Methionine in C57BL/6J Mice

**Figure 1:**
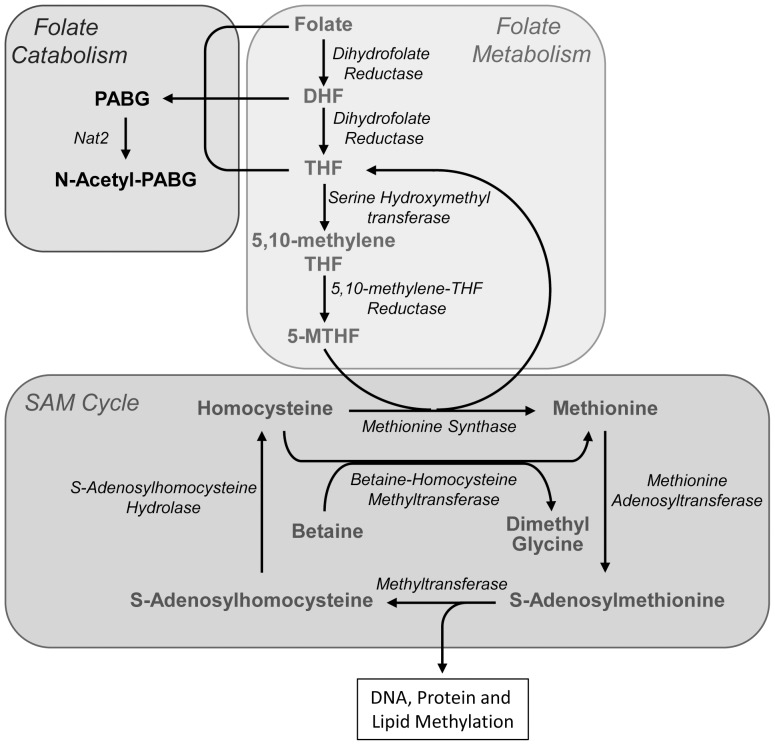
Biochemical pathways for the interaction of folate metabolism and the *S*-adenosylmethionine (SAM) cycle. Dietary folate is metabolized to 5-methyltetrahydrofolate (5-MTHF), which is used by methionine synthase for the conversion of homocysteine to methionine. Methionine can also be synthesized from betaine, especially in liver tissue. Catabolism of the folates by cleavage of the C9-N10 bond produces *p*-aminobenzoylglutamate (PABG) and pterin (not shown). Murine Nat2 (and human NAT1) acetylates PABG to *N*-acetyl-PABG, which is a major folate metabolite found in the urine.

5-MTHF is the predominant folate species in both humans and rodents [1], [13], [20]. It is utilized in the conversion of Hcy to methionine, which is a precursor for the synthesis of SAM. Consequently, 5-MTHF is essential for maintenance of the SAM cycle and one-carbon metabolism (Fig. 1). Several components of the SAM cycle have demonstrated gender differences. For example, female A/J and C57BL/6J mice have approximately 50% higher circulating Hcy than male mice [21]. Similarly, female rats exhibited higher hepatic glycine *N*-methyltransferase, an enzyme that regulates SAM levels, compared to male animals [22].

**Figure 2:**
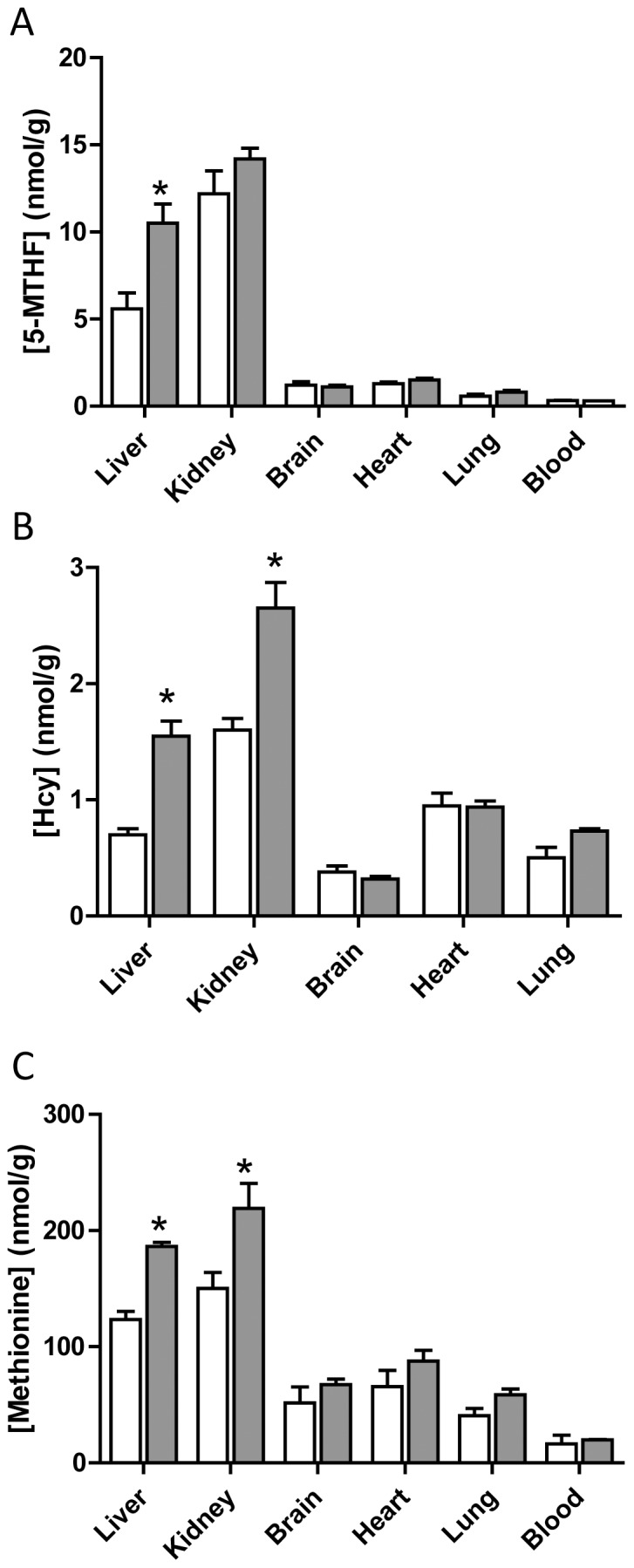
Tissue concentrations of 5-MTHF (A), Hcy (B) and methionine (C) in C57BL/6J mice. The open bars are male tissue samples while the solid bars are female tissue samples. Results are mean ± SEM, n = 3. Asterisk indicates significant difference compared to respective male concentrations (p<0.05).

We measured the concentrations of the SAM precursors, 5-MTHF, methionine and Hcy, in tissues from male and female wild-type C57BL/6J mice. The level of all 3 metabolites was highest in the liver and kidney compared to other tissues examined (Fig. 2). In the liver from female animals, 5-MTHF, Hcy and methionine levels were 50 to 100% higher than in male livers (Fig. 2). Similarly, both Hcy and methionine levels were higher in female kidney. No gender-based differences were seen in brain, heart, lung or blood tissue. These results show that the precursors for SAM biosynthesis are elevated in the liver of female mice compared to male mice.

### Tissue Levels of SAM and SAH in Wild-type Mice

**Figure 3:**
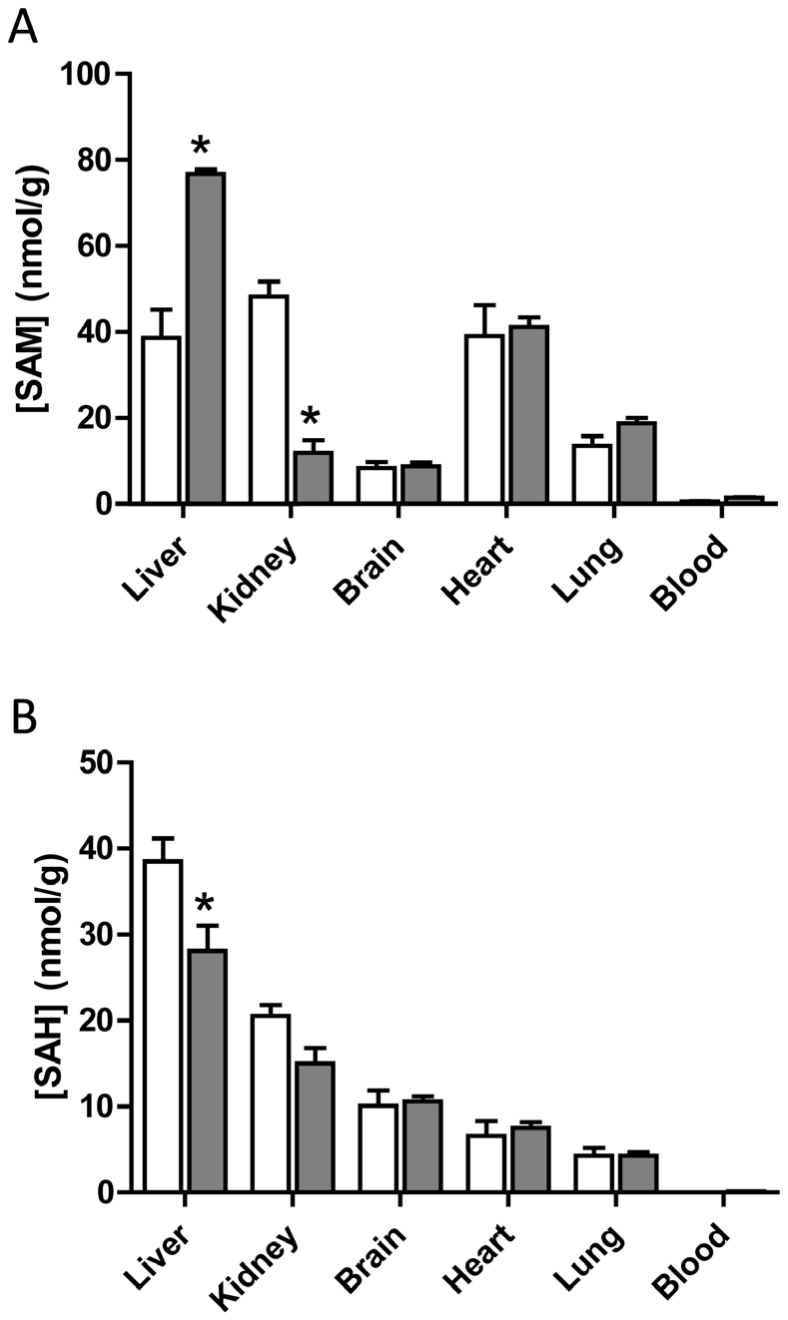
Tissue concentrations of SAM (A) and SAH (B) in C57BL/6J mice. The open bars are male tissue samples while the solid bars are female tissue samples. Results are mean ± SEM, n = 3. Asterisk indicates significant difference compared to respective male concentrations (p<0.05).

Consistent with the elevated levels of 5-MTHF, Hcy and methionine, SAM concentrations were significantly higher in female livers compared to males (Fig. 3). This was accompanied by a decrease in SAH, which suggests methylation turnover may be lower in female liver tissue. Surprisingly, SAM concentrations in the female kidney were only 25% of that seen in the male kidney. Again, no differences were seen in other tissues examined.

### Hepatic and Renal Levels of Folate Catabolites in Wild-type Mice

**Figure 4:**
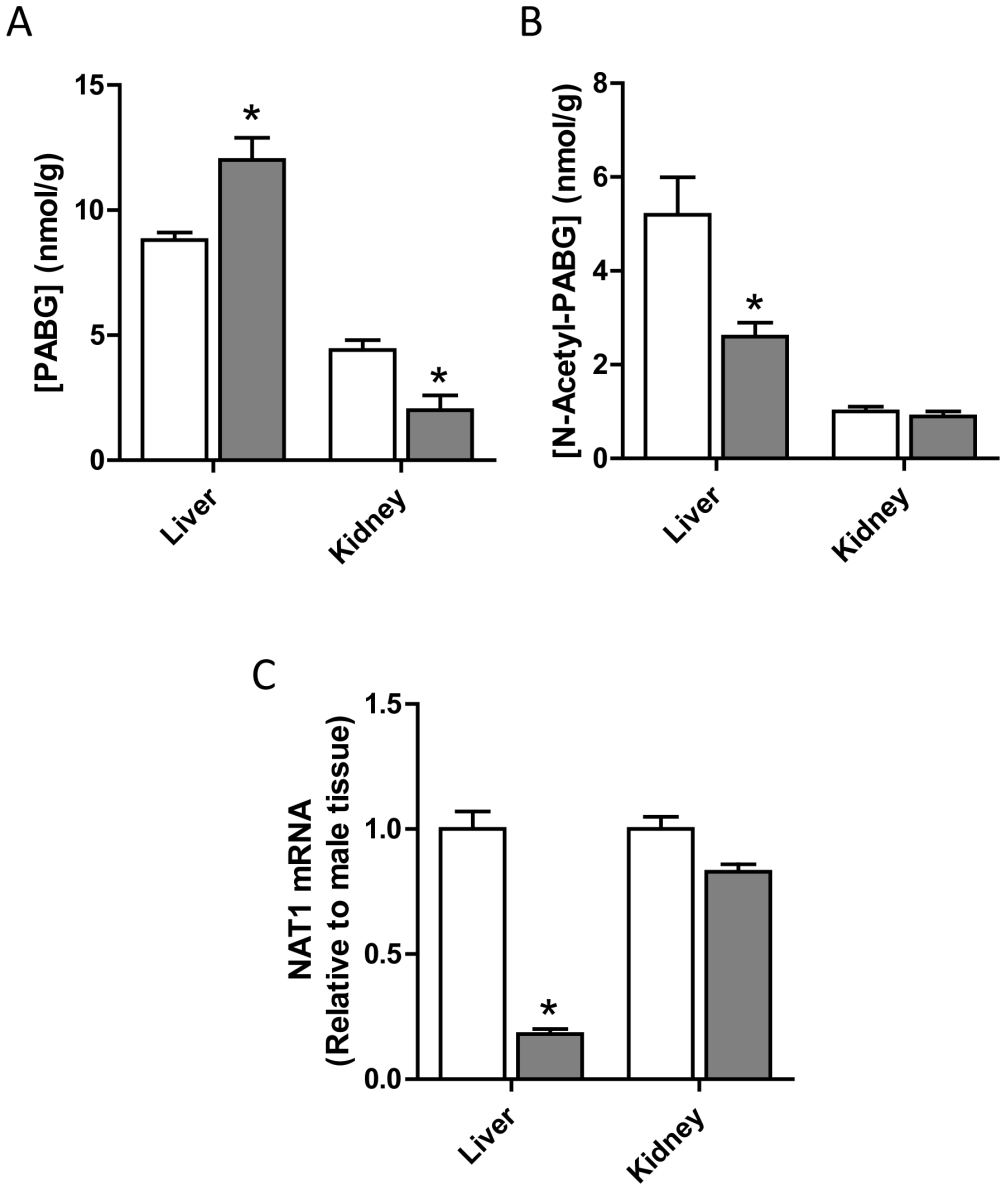
Liver and kidney concentrations of PABG (A) and *N*-acetyl-PABG (B) in C57BL/6J mice. (C) Relative NAT1 mRNA levels determined by qPCR. The open bars are male tissue samples while the solid bars are female tissue samples. Results are mean ± SEM, n = 3. Asterisk indicates significant difference compared to respective male concentrations (p<0.05).

Both PABG and *N*-acetyl-PABG were detected in liver and kidney tissue from wild-type C57BL/6J mice (Fig. 4). Hepatic concentrations for both metabolites were considerably higher than those found in the kidney. In the liver, female mice had higher PABG concentrations than male mice, whereas the opposite was seen for *N*-acetyl-PABG. This suggested that Nat2 may be more highly expressed in the male mouse liver resulting in a larger extent of PABG acetylation. In the absence of a specific murine Nat2 antibody, qPCR was performed on mRNA extracted from liver tissue to detect any gender differences in expression. Male animals exhibited a significantly higher Nat2 mRNA level (approximately 5-fold) compared to female animals, indicative of a higher level of gene expression. In the kidney, there was a slight but significant decrease in Nat2 mRNA in female compared to male mice. The amount of the polyglutamated forms of PABG were below the limit of detection in both tissues examined (data not shown).

### Effect of Nat2 Deficiency on 5-MTHF, Hcy and Methionine Tissue Levels

**Figure 5:**
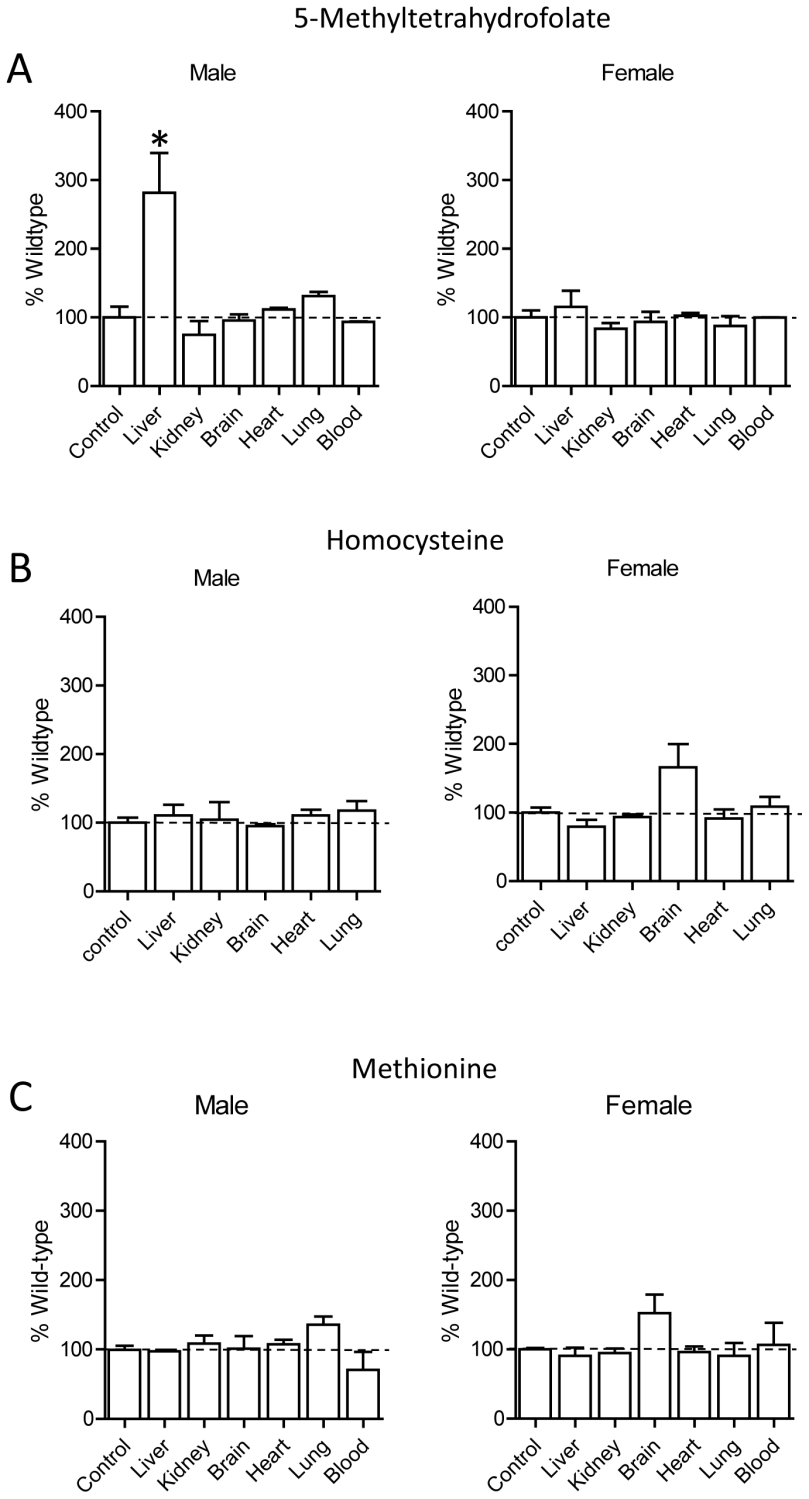
Tissue concentrations of 5-MTHF (A), Hcy (B) and methionine (C) in male and female Nat2−/− C57BL/6J mice. Results are mean ± SEM, n = 3, and are expressed as percentage of wild-type concentrations. Asterisk indicates significant difference compared to respective wild-type concentrations (p<0.05).

In Nat2−/− mice, there was a significant increase in the concentration of 5-MTHF in the liver of male animals but not in female animals, compared to wild-type animals (Fig. 5A). No other differences in 5-MTHF, Hcy or methionine were seen. In the brain tissue of female animals, Hcy and methionine levels were elevated approximately 50% but this was not statistically significant (1-way ANOVA, p>0.05).

### Effect of Nat2 Deficiency on SAM and SAH Tissue Levels

**Figure 6:**
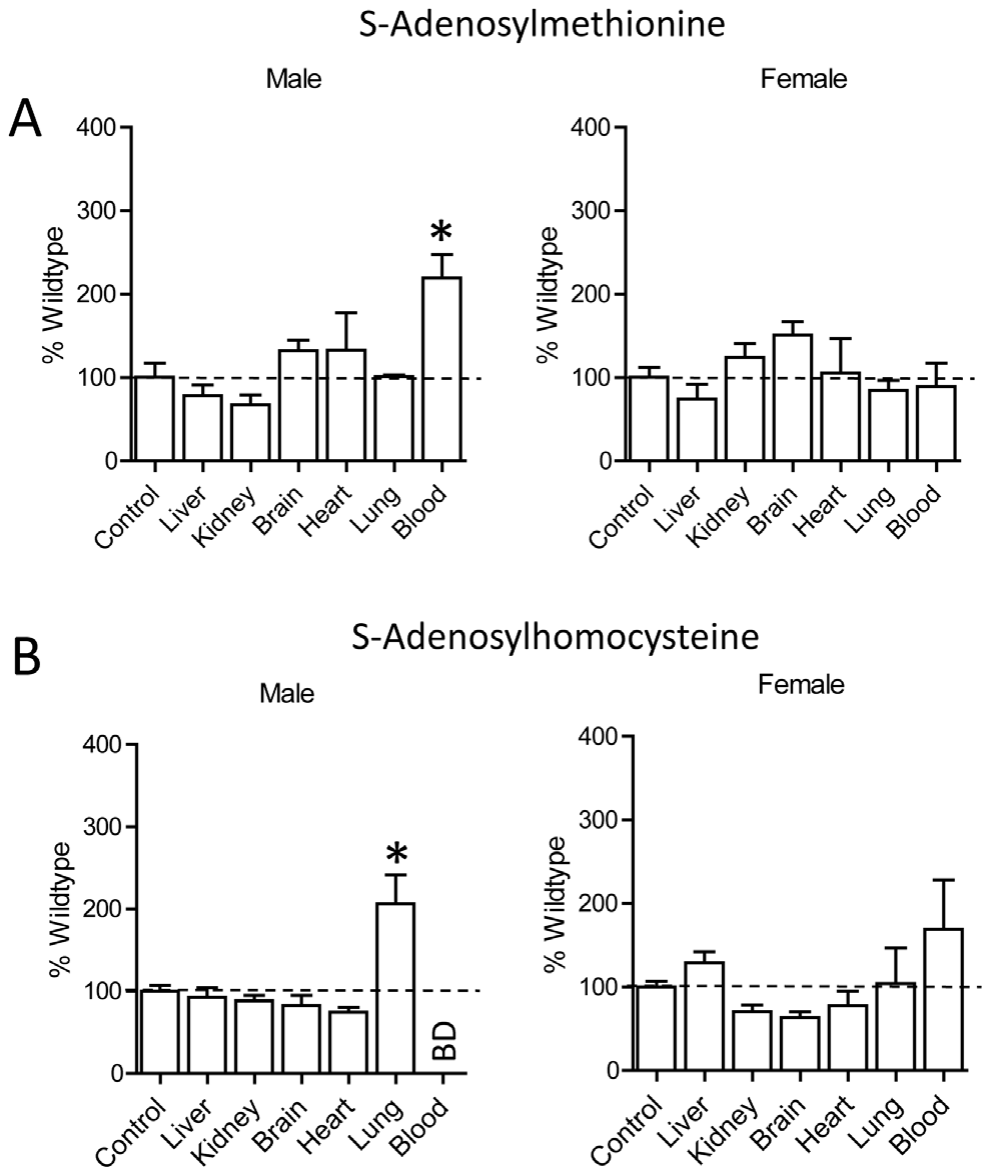
Tissue concentrations of SAM (A) and SAH (B) in male and female Nat2−/− C57BL/6J mice. Results are mean ± SEM, n = 3, and are expressed as percentage of wild-type concentrations. Asterisk indicates significant difference compared to respective wild-type concentrations (p<0.05).

The concentration of SAM in the blood of male Nat2−/− mice was more than twice that seen in wild-type mice (Fig. 6). There was also a significant increase in SAH in the lung of male Nat2−/− animals. By contrast, there were no significant changes in the concentration of either SAM or SAH in the tissues from female Nat2−/− mice compared to wild-type.

### Effect of Nat2 Deficiency on Hepatic and Renal Levels of Folate Catabolites

**Figure 7:**
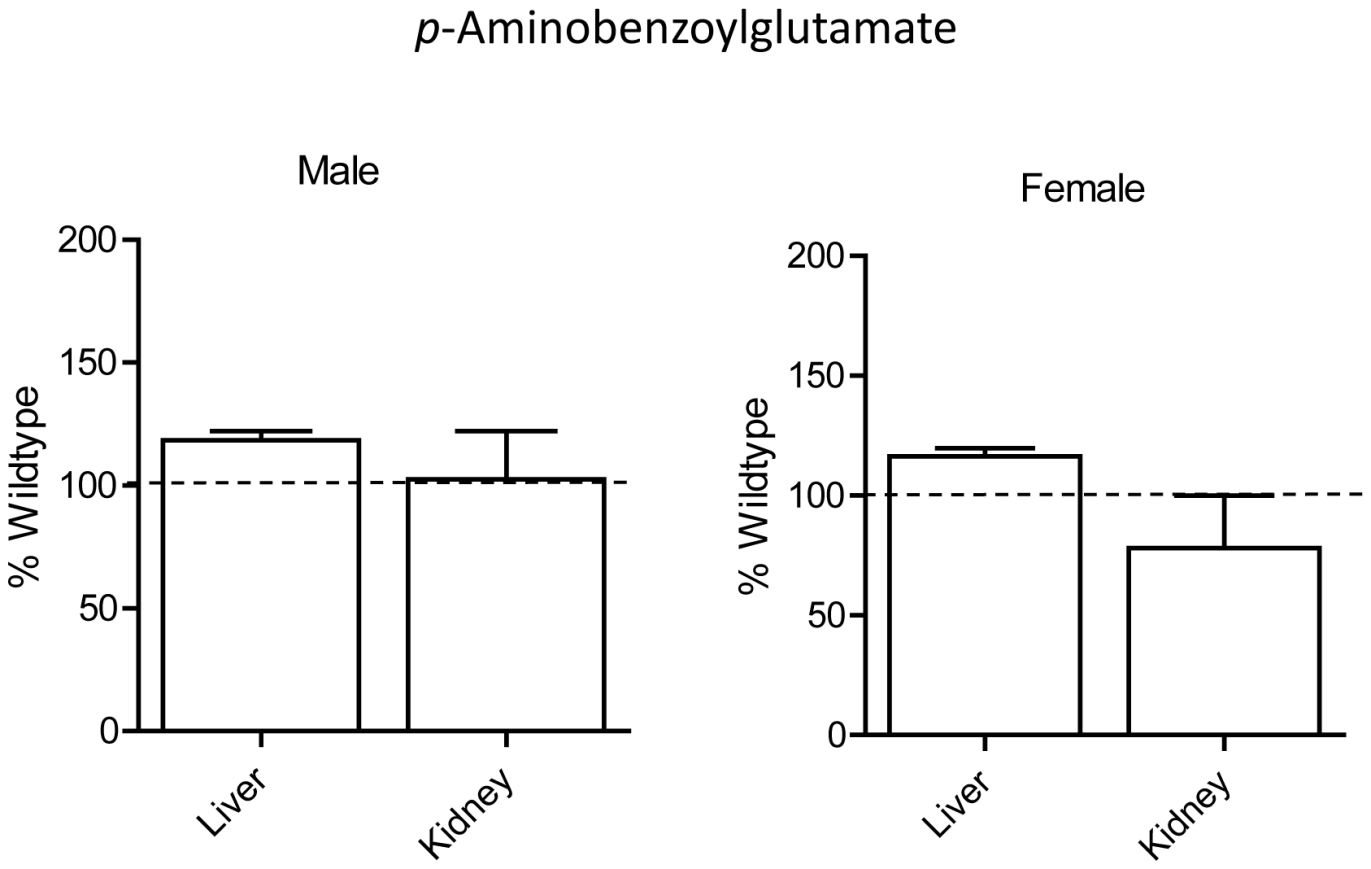
Liver and kidney concentrations of PABG in male and female Nat2−/− C57BL/6J mice. Results are mean ± SEM, n = 3, and are expressed as percentage of wild-type concentrations.

In Nat2−/− mice, *N*-acetyl-pABG was undetectable in both the liver and kidney samples. This is consistent with previous reports [18] and confirms the essential role of Nat2 in the acetylation of PABG *in vivo* (data not shown). Surprisingly, the concentration of PABG in liver and kidney were similar in Nat2−/− mice compared to wild-type mice (Fig. 7). This was true for both male and female animals. These results suggest that Nat2 activity is required for the acetylation of PABG but it is not essential for regulating PABG tissue concentrations, at least in the liver and kidney of C57BL/6J mice.

## Discussion

In the present study, tissue concentrations of the folate precursor to the SAM cycle, 5-MTHF, along with methionine, SAM, SAH and Hcy were evaluated in C57BL/6J mice. These metabolites regulate methylation reactions, including epigenetic control of gene expression, by controlling the levels of SAM in the cell. There is evidence that the folate-SAM cycle pathway varies with gender [21], [23]. In the liver of female mice, 5-MTHF, methionine, SAM and Hcy were significantly elevated compared to male liver tissue. Recently, Chew et al showed that betaine was utilized to a greater extent in methionine synthesis (Fig. 1) in the female BALB/c mouse liver compared to that in male liver [24], which could explain the elevated SAM cycle metabolites in the female liver. Since female mice also show higher DNA methyltransferase activity in the liver [25], the results suggest that female mouse liver may have a higher methylation capacity compared to male animals. In the kidneys, 5-MTHF, methionine and Hcy were significantly higher in females, but unexpectedly, SAM levels were only 25% of that in male tissue. The physiological reason for this difference is currently unknown. There were no differences in any of these metabolites in brain, heart or lung tissue.

Folates are metabolized along a number of pathways. A major catabolic pathway is the formation of PABG following cleavage of the C^9^-N^10^ bond. PABG is readily acetylated by Nat2 to form *N*-acetyl-PABG, which is then excreted in the urine. Previous studies with Nat2 null mice showed no evidence of N-acetyl-PABG formation in the absence of Nat2, indicating its essential role in PABG acetylation [18]. We also observed an absence of *N*-acetyl-PABG following Nat2 gene deletion. However, somewhat surprisingly, there was no change in PABG concentrations in either liver or kidney tissue from male or female animals. These results indicate that PABG levels are not directly regulated by Nat2 activity *in vivo* and suggest that acetylation is of little or no consequence in PABG homeostasis.

The deletion of Nat2 in C57BL/6J mice produced few changes in the folate-SAM pathway. A notable exception was a significant increase in hepatic 5-MTHF, which was only seen in the male animals. Following gene knockout, the male liver 5-MTHF levels increased to that seen in wild-type female mice. Interestingly, female liver tissue expressed much lower levels of Nat2 compared to males. Taken together, these results suggest there is an inverse relationship between hepatic 5-MTHF and Nat2 activity.

In summary, the current study has demonstrated gender differences in the folate-SAM cycle in murine liver and kidney tissue. Deletion of Nat2 selectively affected 5-MTHF in male liver tissue but had no effect on the concentrations of its putative endogenous substrate PABG. It should be noted that the tissues used in the present study were harvested from animals raised under normal conditions where no phenotypic differences between the strains is evident. More pronounced changes in folates and the SAM cycle may be evident under folate deficiency or following other stresses that alter the folate-SAM cycle pathway.
